# Employment and People with Non Communicable Chronic Diseases: PATHWAYS Recommendations and Suggested Actions for Implementing an Inclusive Labour Market for All and Health in All Sectors

**DOI:** 10.3390/ijerph15081674

**Published:** 2018-08-07

**Authors:** Matilde Leonardi, Chiara Scaratti

**Affiliations:** Neurology, Public Health, Disability Unit-Fondazione IRCCS Istituto Neurologico Carlo Besta, 20133 Milan, Italy; chiara.scaratti@istituto-besta.it

## 1. Employment and People with Non Communicable Chronic Diseases: The State of the Art

The increasing prevalence of chronic diseases among the European working age population, as well as the implications for the individual and societal levels, underline the need for policy guidelines and recommendations targeting the effective inclusion and/or reintegration of persons with chronic diseases, also known as non-communicable diseases (NCDs), in the workplace, in addition to the definition of actions that allow people to operationalize these recommendations. Chronic diseases are defined by the World Health Organization (WHO) as diseases that are not passed from person to person, and which generally have a long duration and slow progression.

Recent data from 27 EU member states showed that about one quarter of the working age population (23.5%) had a chronic disease, while 19% reported having long-standing health issues. Work and health are interrelated in many ways. The ageing of the working population combined with the dramatic low employment rates of persons with chronic diseases (PwCDs) is an indicative depiction of this particular relation [1].

The aim of this special issue on employment and chronic conditions reflects the results of three years of work performed by the consortium of the EU project PATHWAYS—Participation to Healthy Workplaces and Inclusive Strategies in the Work Sector (www.path-ways.eu), which aimed at exploring the current situation of strategies targeting employment inclusion and re-integration of PwCDs in Europe and across European countries, as well as the opportunities to promote their participation in the labour market. This work was conducted from the perspective of national and European level stakeholders from all sectors, including policy makers and civil society. Given the variety of sectors involved in the issue of employment and NCDs, the PATHWAYS project underwent a structured evaluation process and a clear monitoring methodology which was developed and tested throughout the whole project and that is suggested as essential in every multidisciplinary research project involving different stakeholders in diverse socio-environmental and political conditions [2].

Work, as an important life domain, has a positive impact on health and functioning in PwCDs and an important share of PwCDs who are unemployed due to health-related problems wish to return to work and regain participation in this important life domain. To overcome this contradiction, the European Union has invested considerable resources in the field. A considerable number of strategic papers and reports have stressed the need to foster the employability of PwCDs. PATHWAYS faced the challenge of providing evidence-informed recommendations regarding strategies for the integration and re-integration of PwCDs into work. 

**Figure 1:**
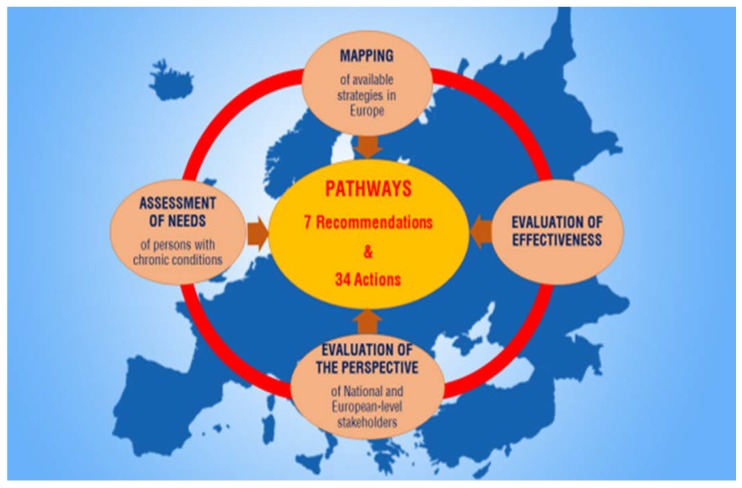
PATHWAYS focused on the situation of PERSONS WITH CHRONIC CONDITIONS and EMPLOYMENT and had four research streams.

PATHWAYS had four research streams (Figure 1):

1. The mapping of professional integration and reintegration strategies for PwCDs, available at European and national levels
2. The evaluation of strategies (policies, systems, and services) targeting the professional reintegration of PwCDs in Europe
3. The assessment of specific employment-related needs of PwCDs
4. The National and European-level stakeholders’ surveys on the implementation of reintegration strategies for PwCDs

The main challenge for the PATHWAYS project was being able to propose a novel paradigm for Europe where employment is the Trojan horse for a broader societal reform of the welfare systems, able to take into account the rising and increasing burden of NCDs in ageing societies.

As highlighted in the paper of Sabariego and colleagues [3], people with chronic diseases face a variety of important problems in performing their every-day lives and in participating in society, with work being one of the major areas affected. Chronic diseases, such as low back pain, migraine, diabetes, and depression, all have peaks in disease onset that are in the most productive years of adults’ working lives. NCDs are an important cause of absence from work, but are also linked to high levels of presenteeism, where an employee remains at work despite experiencing symptoms that result in lower productivity.

As presented in the comprehensive evaluation of regulations, laws and acts in different European countries performed by Scaratti and colleagues [4], following the definition of disability of the Convention on the Rights of Persons with Disabilities (PwD) and due to the burden they experience in daily life, PwCDs can be well considered under the definition of PwD. This fact has been corroborated by diverse studies and also becomes clear if we look at the Global Burden of Disease Study [5], which overwhelmingly identified NCDs, often chronic conditions, as those mostly associated with disability. In fact, most people who receive disability benefits in Europe have chronic diseases. The work of Scaratti et al. presented in this issue is an evaluation of employment at policies, systems, and services levels, and it shows that in many countries, strategies targeting persons with disabilities do not necessarily address the needs of patients with chronic diseases and mental health conditions since the employment needs of these groups are not always the same. Identifying the work-related needs of PwCDs and developing tailored interventions, they conclude, may be important for the secondary prevention of illness becoming chronic.

The labor market is undergoing profound changes and societies will increasingly be in need of adequate information on which determinants of employment could be addressed to enhance employment rates of European citizens. The paper from Leonardi and colleagues [6] reports information on health and epidemiological factors related to unemployment status from a wide sample of citizens from Finland, Poland, and Spain collected within the EU COURAGE in Europe project. The paper reports that studies addressing the impact of health on employment status generally found that people with worse health, with poor mental health or chronic health conditions, also show lower employment rates. Self-rated health outcomes were found in COURAGE to be a factor pertinent for unemployment. The COURAGE results show that some of these factors can be addressed with short-medium term interventions that focus on health promotion and health prevention strategies: for example, interventions aimed at promoting physical activity and healthy behaviors, tackling depression or promoting education, in particular for those who are younger.

In line with the issue that people with mental health problems in the work sector are facing problems that need a more tailored approach is the paper from Muñoz-Murillo and colleagues [7], which highlights the complexities of the implementation of employment strategies (job access and return to work) for people with mental health problems. Job access strategies seem to improve employment outcomes, while the effectiveness of return to work strategies remains unclear. In this framework, the involvement and commitment of physicians, employment specialists, and employers, together with the employees’ capacity for self-care, seem decisive for employment re-integration success.

What clearly emerged during PATHWAYS’ work is that European stakeholders are currently looking for innovative strategies to improve the integration and reintegration of PwCDs in work, but also that the direct involvement of PwCDs is crucial. Esteban and colleagues [8] made a review that synthesized 24 qualitative studies exploring the views and experiences of PwCDs in European countries regarding strategies to facilitate and manage their integration and reintegration in work life. What emerged from several studies is that PwCDs want to work or want to return to work. Gaining work or being involved in work are in fact important aspects of people’s lives and contribute to the financial security of persons and their relatives, as well as social participation. This review informs stakeholders about key elements of experiences and views of PwCDs, which must be taken into account when implementing strategies and programs supporting professional integration and reintegration of PwCDs with the final goal of being effective and well-accepted. With its focus on qualitative studies, this review complements existing reviews, also presented in this issue.

The ability to maintain a job or return to work does not only depend, however, on the health conditions, but is importantly influenced by a person’s physical, social, attitudinal, and political environment. Adapting the working environment to the needs of persons with chronic health conditions is a task that employers and politicians have to also consider in light of an aging population and an increase of chronic health conditions that European countries will face during the next decades. However, knowledge of factors that have a positive and negative impact on work life, as well as of the work-related needs of persons with NCDs, is necessary to identify or develop interventions that can support those people maintaining a job or returning to work after a long absence. Taking this into account, PATHWAYS performed a quantitative study on 688 participants with six chronic health conditions in nine countries representing four welfare systems in Europe (Continental, Mediterranean, Post-communist, and Scandinavian). Employment needs were, for the purpose of the project, defined according to the framework of the WHO International Classification of Functioning, Disability and Health (ICF) [9], as the modifiable environmental and/or personal factors that hinder (barriers) or/and facilitate (facilitators) people with chronic health conditions to participate in the labor force and to perform work activities in a similar way as people without chronic conditions. Quantitative results presented in the paper from Ávila and colleagues [10] showed that “Raising awareness of what is to live with a chronic health condition in the workplace” was the area perceived as more favorable and, on the contrary, “Giving the company the possibility lo legally terminate the job in case productivity decreases due to chronic condition” was rated as unfavorable/very unfavorable by 75% of participants. The types of employment needs were different across the social welfare systems, but did not vary among the different chronic health conditions groups. A qualitative part including open-ended questions was added to this survey, and the work from Foitzek and colleagues [11] presents the qualitative analysis of the answers of 487 participants out of the 688, showing the needs and the factors that have a negative or positive impact on the work lives of persons with chronic health conditions.

The majority of participants named work-related aspects (such as career development, stress at the workplace, work structure and schedule, as well as workload), support of others, and attitudes of others as being the factors positively and negatively impacting their work lives the most. The study shed light on the importance of changing the attitudes of supervisors and co-workers to counteract the stigmatization of persons with chronic health conditions in the workplace.

The results of the quantitative and qualitative studies on the needs of people with NCDs in the workplace are also supported by the review from Sabariego and colleagues [3], which found that positive changes in employment status, return to work, and sick leave outcomes of persons with chronic diseases and with disability in general can be facilitated with graded sickness-absence certificates; part time sick leave; early interventions; disability evaluation followed by information and advice; and multidisciplinary, coordinated, and tailored return to work interventions. Additionally, the review found a positive association between the co-existence of (a) active labour market policies to promote employment and (b) passive support measures (e.g., pensions) and the probability of finding a job.

## 2. Conclusions

This special issue on employment and chronic conditions could not have come at a better time. The number of people with chronic conditions is increasing worldwide but, despite this, they continue to be unknown in major areas of health and health-related sectors. The employment sector is undergoing profound changes and societies will increasingly be in need of adequate information on which determinants of employment could be addressed to enhance the employment rates of all European citizens. Many of the factors that influence the wellbeing of workers relate to the social environment at work, such as the working conditions, style of management, working culture, and levels of support, as well as job security. A healthy workplace involves creating an environment that is supportive of all the aspects of work, recognizing the potential of the workplace to promote workers’ health and wellbeing, and reducing the negative impacts of work-related stress.

**Figure 2:**
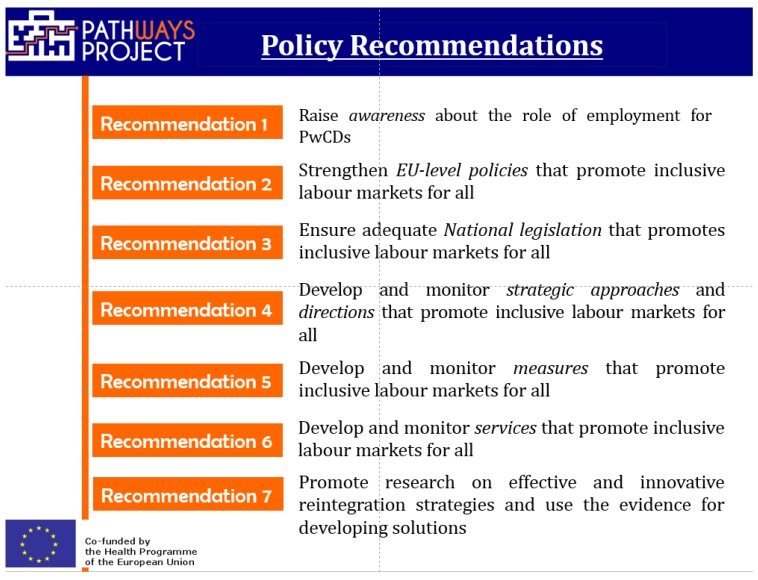
Pathways Policy Recommendations for NCDs and employment.

We hope that this special issue will promote a greater awareness worldwide of the barriers to the implementation of effective interventions, in particular for smaller enterprises and public agencies with less resources and knowledge to manage the issues that people with NCDs face in the work sector. PATHWAYS’ results stress the importance to adapt the workplace and the environment according to the needs of persons facing chronic health conditions and to enable these persons to maintain a job or return to work in the long run. In light of an aging working population and an increase of chronic health conditions in European countries, this will be one of the most important tasks that employers and politicians will have to take into account during the next decades. The PATHWAYS project provides first-hand evidence for recommending strategies of integration and reintegration at work for persons with chronic health conditions in European countries, and is based on solid methodology that also includes an extensive evaluation of national and European stakeholders’ perspectives and needs [1]. PATHWAYS Policy Recommendations for the employment of people with NCDs (Figure 2) have been developed to support both people with NCDs, who have to be recognized as a population who needs accessible and inclusive pathways for improving and maintaining their own health and their jobs, as well as the employment sector, which needs to be a facilitator for all but also requires support in learning how to do this (PATHWAYS Policy recommendations are included in the Supplementary Material). These recommendations and the actions that operationalize them also provide the base for actions implemented in the Join Action CHRODIS Plus, in the Employment and chronic diseases work-package (http://chrodis.eu).

The real challenge, for an approach where health is considered in all sectors, is to develop an employment sector able to adapt to each person’s needs and that does not constrain persons to adapt to employment that is a barrier to their health. An inclusive labour market for all will greatly support health for all.

## Supplementary Materials

The following are available online at http://www.mdpi.com/1660-4601/15/8/1674/s1, File 1: PATHWAYS Policy recommendations.
