# Supplementary material for: Employment and People with Non Communicable Chronic Diseases: PATHWAYS Recommendations and Suggested Actions for Implementing an Inclusive Labour Market for All and Health in All Sectors
Source: Int J Environ Res Public Health. 2018 Aug 7;15(8):1674. doi: 10.3390/ijerph15081674 (PMC6121237; doi:10.3390/ijerph15081674)
Supplement: Supplementary file 1 [file ijerph-15-01674-s001.pdf]

Co-funded by
the Health Programme
of the European Union

7 RECOMMENDATIONS & 34 ACTIONS

Pathways for inclusive labour markets in Europe:

Employment for all Persons with Chronic Diseases

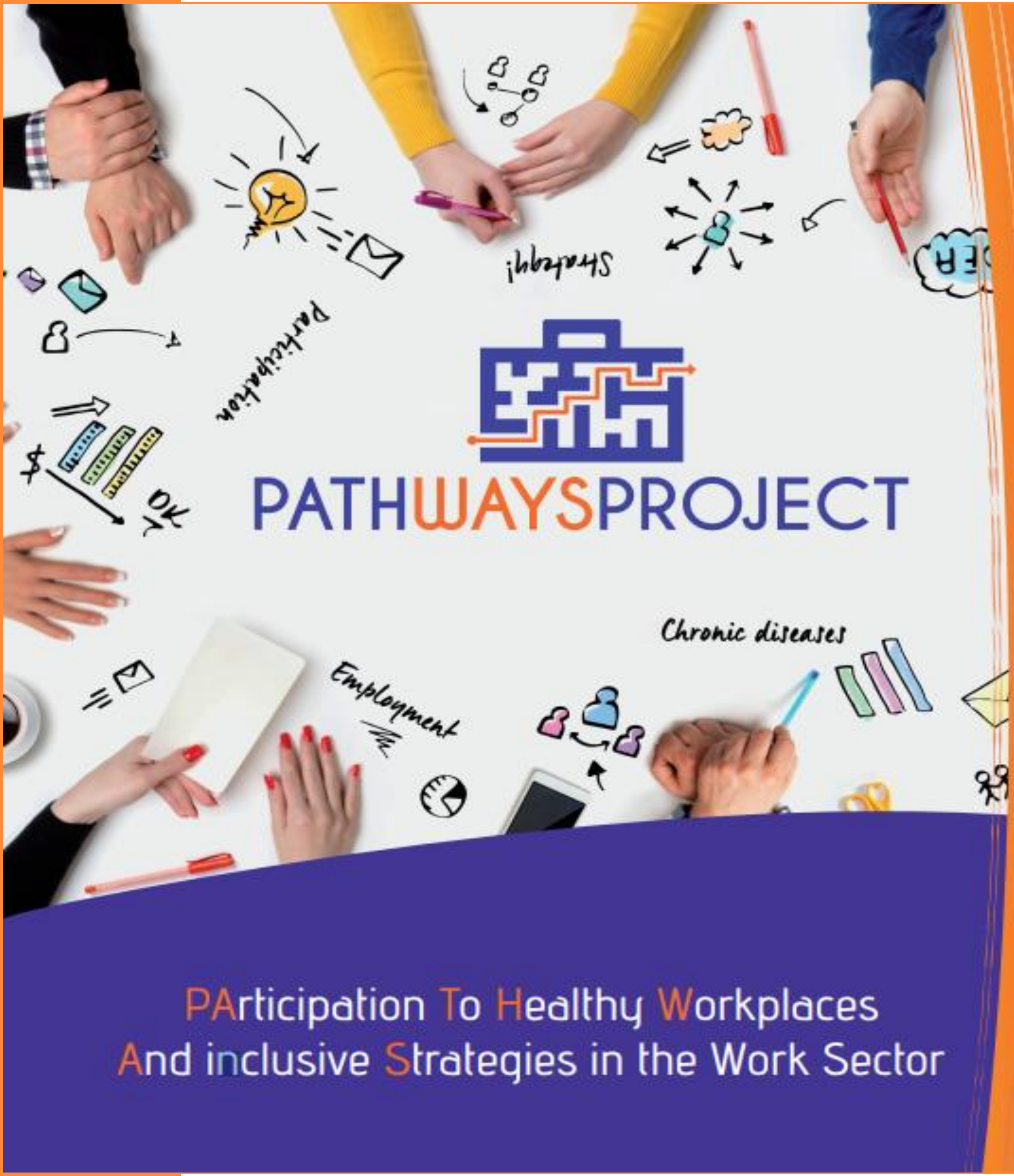

www.path-ways.eu

PATHWAYS project has received funding from the European Union's Health Programme 2014-20202

Grant agreement n. 663474 (2015-2018)

7 Recommendations & 34 Actions

Pathways for inclusive labour markets in Europe

Employment for all Persons with Chronic Diseases

The 7 Recommendations and **corresponding 34 Actions** for reaching an inclusive labor market in all European Countries were developed in the scope of the EU-funded 3-year international project *"Participation to Healthy Workplaces and Inclusive Strategies in the Work Sector"*, **PATHWAYS,** taking into account: the availability of policies, systems and service strategies and their effectiveness, the perspective of national and European stakeholders, and the met and unmet employment needs of persons with chronic diseases, PwCDs.. CDs, also known as noncommunicable diseases (NCDs)according to the definition of WHO adopted in PATHWAYS project, are not passed from person to person, are of long duration and generally slow progression.

PATHWAYS focused on the situation of PERSONS WITH CHRONIC CONDITIONS and EMPLOYMENT and had four research streams:

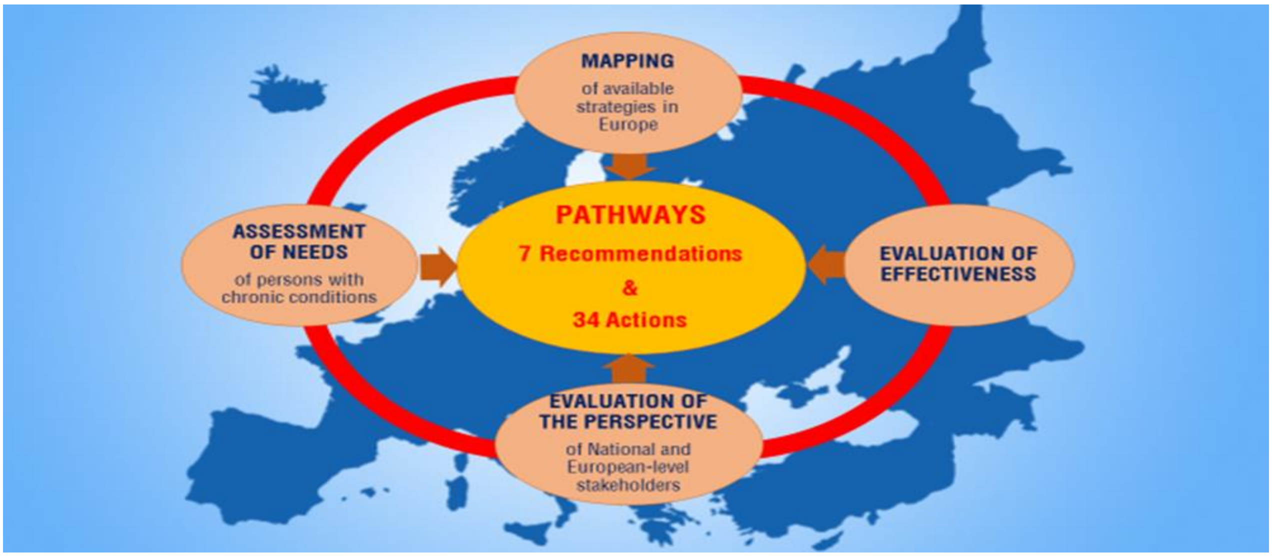

In all research streams, PATHWAYS focused on chronic diseases selected on their burden and contribution to years lost due to disability: mental health conditions, neurological diseases, metabolic disorders, musculoskeletal disorders, respiratory diseases, cardiovascular diseases and cancer.

1. MAPPING

Strategies for inclusion, integration and reintegration of PwCDs at both European and national levels, implemented at policies, systems, and services levels were mapped. *(WORKPACKAGE 4)*

2. EVALUATION OF EFFECTIVENESS

The effectiveness of policies, systems and services strategies targeting professional integration and reintegration of persons with chronic diseases was evaluated in a systematic review. *(WORKPACKAGE 5)*

3. ASSESSMENT OF EMPLOYMENT NEEDS OF PWCDs

Persons with six different chronic conditions living in nine European countries participated in an *e*-survey reporting the factors they perceived as favorable or unfavorable to obtain and retain employment. *(WORKPACKAGE 6)*

4. EVALUATION OF NATIONAL AND EUROPEAN STAKEHOLDERS' PERSPECTIVES

The perspectives of European and National-level stakeholders – policy makers, unions, professionals and employers – on existing strategies for work re-integration of persons with chronic diseases were explored using an online survey. *(WORKPACKAGE 7)*

Co-funded by the Health Programme of the European Union

PATHWAYS
7 POLICY RECOMMENDATIONS

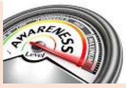

1# Raise *Awareness* about the role of employment for persons with chronic diseases

The needs of PwCDs in the area of integration, inclusion and reintegration into the labour sector must be prioritized through targeted actions where biological, psychological, social factors and their complex interactions are considered. Health in all policies implies that Governments have a responsibility for the health of their people. This can only be fulfilled by the provision of adequate health and social measures including awareness raising and information about chronic diseases and their impact on employment as well as the impact of employment on PwCDs.

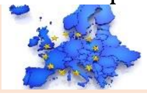

2# Strengthen *EU-Level Policies* that promote inclusive labour markets for all

Reduced unemployment, social equality and higher labour market participation are the main priorities of the EU's 2020 Disability Strategy. The European Commission has highlighted the importance of building an inclusive labour market for all of all ages, , where all working-age and especially vulnerable and disadvantaged persons, should be given an equal opportunity to enter and remain.

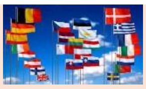

3# Ensure adequate *National legislation* that promotes inclusive labour markets for all

In many EU-countries persons with specific chronic health conditions can receive support in employment only if their condition is legally recognized as a "disability". Such frameworks, however, create an "invisible" category of working-age PwCDs that are "not disabled" and will not be eligible for national policies, although they face obstacles to participate in the open labour market.

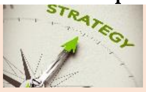

4# Develop and monitor *strategic approaches* and *directions* that promote inclusive labour markets for all

The achievement of the inclusion and full participation of each person in the employment sector necessitates strategic actions on the part of employees, employers and all other stakeholders involved, in order to combine incentives and obligations that together achieve inclusion. A shift in attitudes of all parties and a genuine willingness/commitment of all to eliminate workplaces' restrictions for all, thus including PwCds, is a must-factor in the changing process.

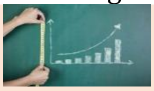

5# Develop and monitor *measures* that promote inclusive labour markets for all

Promotion of sustainable employment of PwCDs involves the development of measures that take into account PwCDs' need for job security, employment support and reasonable accommodation as well as employers' need for economic sustainability.

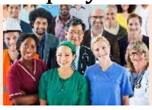

6# Develop and monitor *services* that promote inclusive labour markets for all

Biopsychosocial factors that limit employment reintegration or integration of PwCDs are diverse and multidimensional. They include personal, societal and environmental work-related parameters. Thus, different types of services and supports to address this heterogeneity are needed.

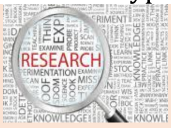

7# Promote *research* on effective and innovative strategies and use the available evidence for developing solutions for inclusive employment

The development of agreed study protocols and the assessment of strategies should be at the centre of the governance structure in order for policy makers to collect and rely on valid and reliable information for establishing and monitoring the effects of existing strategies for work integration and reintegration. This contributes to evidence-based decisions and policies.

1# Raise *Awareness* of the role of employment for persons with chronic diseases

PATHWAYS FINDINGS

MAPPING

- ✓ In many European countries awareness of the role of employment for persons with noncommuni diseases (NCDs), also known as chronic diseases, CDs, is still lacking.

EVALUATION OF EFFECTIVENESS

- ✓ Stigma and discrimination were identified as factors limiting the effectiveness of strategies aiming to improve work reintegration of PwCDs.
- ✓ Effective strategies are highly affected by the way disclosure of illness and the need for social support is handled in the work environment.

ASSESSMENT OF NEEDS

- ✓ PwCDs, regardless of the type of health condition, diagnosis or symptoms, highlighted as part of their most important needs: awareness raising for co-workers, human resources and employers on health conditions and their specific management.

EVALUATION OF THE PERSPECTIVE OF STAKEHOLDERS

- ✓ Stigma, discrimination and the lack of awareness were considered barriers for the effective implementation of inclusive working strategies by National and EU level stakeholders
- ✓ Awareness raising and training for staff and management were considered of crucial importance by both National and EU level stakeholders for promoting labour market participation of PwCDs

	PATHWAYS RECOMMENDED ACTIONS	Level
1	**DESIGN DISSEMINATION STRATEGIES** to raise awareness and inform society about chronic diseases and their impact on employment. Information should cover all levels of society to create a competent and aware environment.	**Policy**
2	**DEVELOP SERVICES PROMOTING AWARENESS** raising and training for service providers and human resources managers to ensure a better understanding of PwCD's needs.	**Policy, Services**
3	**INFORM EMPLOYERS** about the benefits of employing and retaining PwCDs at work.	**Systems, Services**
4	**INFORM EMPLOYEES** with chronic diseases about their work-related rights and the availability of support and/or services.	**Systems, Services**

2# Strengthen EU-level policies that promote inclusive labour markets for all

PATHWAYS FINDINGS

MAPPING

- ✓ Employment of PwCDs is regulated to a large extent through:
  a. policy frameworks for the employment of persons with disabilities;
  b. policy frameworks for employment activation and inclusion in the labour market;
  c. a number of individual reports and actions that target specific chronic conditions

EVALUATION OF EFFECTIVENESS

-

ASSESSMENT OF NEEDS

-

EVALUATION OF THE PERSPECTIVE OF STAKEHOLDERS

- ✓ EU stakeholders indicated: a) modest availability of policy directions and legislation regulating employment reintegration; b) lack of specific and adequate policy directions on unemployment reduction and on promotion of employment reintegration of PwCDs
- ✓ EU stakeholders considered policy implementation as being significantly limited due to: a) inadequate and ineffective coordination among agencies involved; and b) lack of monitoring and evaluation of existing strategies

	PATHWAYS RECOMMENDED ACTIONS	Level
1	**PUT IN PLACE POLICY PROVISIONS** that target employment activation and participation of all, including PwCDs, in accordance with EU policies for equal access to employment.	**Policy**
2	**DEVELOP COUNTRY-TAILORED POLICIES** adapted to the various employment needs across EU countries and across different EU social welfare models.	**Policy**
3	**PROMOTE HEALTH AT WORK** taking into account challenges faced by labour markets due to an aging workforce, later retirement and increasing prevalence of chronic diseases.	**Policy, Systems**
4	**ENACT POLICY PROVISIONS** that focus on the *capacity* to work of PwCDs.	**Policy**
5	**PROMOTE THE “BIOPSYCHOSOCIAL APPROACH”** to health in all sectors, regarding the development of healthcare interventions for PwCDs and the evaluation of their results.	**Policy**
6	**CONSIDER “EMPLOYMENT” AS A POSITIVE OUTCOME** of all health-investment decision making.	**Policy, Systems**

3# Ensure adequate National legislation that promotes inclusive labour markets for all

PATHWAYS FINDINGS

MAPPING

- ✓ Effective national policies involve the development of adequate flexible, country-tailored national legislation focusing on the right of every person to work and have full participation in employment.

EVALUATION OF EFFECTIVENESS

-

ASSESSMENT OF NEEDS

- ✓ Legislations that allow to be legally protected against being dismissed due to discrimination were reported as very favorable by at least 80% of people. In contrast, legislations that allow legally terminate contracts in case productivity decrease because of health conditions were perceiv very unfavorably by participants suffering from chronic health conditions
- ✓ Differences emerged between PwCDs belonging to countries with different welfare Models, indicating the need for country-tailored actions.

EVALUATION OF THE PERSPECTIVE OF STAKEHOLDERS

- ✓ «Inadequate legislation» was one of the most frequently mentioned barrier hindering the promotion implementation of more inclusive work related strategies.

	PATHWAYS RECOMMENDED ACTIONS	Level
1	**DEVELOP A HUMAN RIGHTS-BASED APPROACH** in national employment policies for the re-integration or inclusion of PwCDs in the open labour market. In coherence with UNCRPD art 27. ..”States Parties shall safeguard and promote the realization of the right to work, including for those who acquire a disability during the course of employment, by taking appropriate steps, including through legislation”,	**Policy**
2	**ENACT LEGAL FRAMEWORKS** that promote the combination of active labour market policies to promote employment and passive measures to support PwCDs.	**Policy**
3	**DEVELOP FLEXIBLE LEGISLATIVE FRAMEWORKS** for employment support of PwCDs , not linked only to the presence of a disability certification.	**Policy**
4	**INTEGRATE FLEXIBLE LABOUR MARKET POLICIES** able to promote greater involvement of employers in recruiting and retaining PwCDs at work.	**Policy**
5	**PROMOTE SUSTAINABLE EMPLOYMENT FOR ALL**, including PwCDs in the open labour market.	**Policy, Systems**

4# Develop and monitor strategic approaches and directions that promote inclusive labour markets for all

PATHWAYS FINDINGS

MAPPING

- ✓ Integrated employment support systems that encompass various areas of support (i.e. job-search support, access to training, individualized and specialized support by professionals)

EVALUATION OF EFFECTIVENESS

- ✓ Balance between compensation and activation measures

ASSESSMENT OF NEEDS

- ✓ At least a 70% of participants considered very important to have professional and psychological support during transition phases such as while getting a new job or going back after a longer period of sick leave or after being temporary considered unable to work

EVALUATION OF THE PERSPECTIVE OF STAKEHOLDERS

- ✓ Greater involvement of employers in the process of developing and promoting work reintegration strategies

	PATHWAYS RECOMMENDED ACTIONS	Level
1	**DEVELOP EMPLOYMENT SUPPORT PROGRAMS** that combine incentives and obligations that together achieve inclusion.	**Systems**
2	**PROMOTE REGULATORY COHERENCE** through coordination mechanisms and effective communication among agencies and services dealing with employment re-integration/inclusion of PwCDs. Promote the knowledge of European and international laws and norms, in particular UN Convention for the Rights of People with Disability art.27, on reasonable accommodation, right to work, access to work, prohibition of discrimination	**Systems**
3	**DEVELOP INTEGRATED EMPLOYMENT SUPPORT SYSTEMS** that encompass various areas of social, healthcare, rehabilitation, training and other supports for employment re-integration of PwCDs.	**Systems**
4	**PROMOTE STRATEGIES** that focus on a greater involvement of employers' organizations, of employees' networks and trade unions.	**Systems**
5	**INTEGRATE SPECIALIZED SUPPORT** by professionals to mainstream employment services.	**Systems, Services**

5# Develop and monitor measures that promote inclusive labour markets for all

PATHWAYS FINDINGS

MAPPING

- ✓ Existing measures targeting work reintegration of PwCDs take different forms and orientations while their effectiveness and degree of implementation vary according to the welfare model adopted by different EU-countries.

EVALUATION OF EFFECTIVENESS

-

ASSESSMENT OF NEEDS

- ✓ Physical adaptations of workplace and receiving appropriate education and training to promote professional development were among the most favorable domains reported by PwCDs participating in the Pathways needs assessment survey.

EVALUATION OF THE PERSPECTIVE OF STAKEHOLDERS

- ✓ EU level stakeholders emphasized the need to maintain a balance between protection and activation of PwCDs, and the importance of providing financial supports during participation in employment activation measures.

	PATHWAYS RECOMMENDED ACTIONS	Level
1	**ESTABLISH MEASURES TO MAINTAIN A BALANCE** between employers' commercial interests and job protection for PwCDs.	Systems
2	**DEVELOP MEASURES TO FACILITATE FINANCIAL SUPPORT** for workplace adjustments, adaptations and reasonable accommodation.	Systems
3	**UNDERTAKE MEASURES ENCOURAGING PwCDs TO PARTICIPATE** in employment activation programs, instead of providing only passive benefits (e.g pensions, disability benefits..).	Systems
4	**UNDERTAKE MEASURES THAT SUPPORT THE ECONOMIC SUSTAINABILITY**, competitiveness and commercial activity of social enterprises involving PwCDs.	Systems
5	**RAISE AVAILABILITY OF SUPPORTED EMPLOYMENT PROGRAMS** that report positive employment outcomes.	Systems
6	**PROMOTE MEASURES** that ensure time-off for medical appointments, flexible work routine and job security.	Systems

6# Develop and monitor services that promote participation and an inclusive labour markets for all

PATHWAYS FINDINGS

MAPPING

✓ Research evidence demonstrate the existing gap of adequate and effective services for PwCDs.

EVALUATION OF EFFECTIVENESS

✓ The service-level strategies that were identified as effective by PATHWAYS research were:

- Individual Placement and Support
- A work-focused, early ergonomic and personalized intervention
- Psychological or behavioral strategies
- Multidisciplinary interventions
- Educational training/ re-training

ASSESSMENT OF NEEDS

Regarding the area of employment services two factors were rated as relevant, helpful and favorable by more than 70 % of participants: a) the possibility of establishing positive relationships with health care professionals, social security or employment officers and b) that health services are provided outside typical working hours.

EVALUATION OF THE PERSPECTIVE OF STAKEHOLDERS

✓ EU-level stakeholders highlighted the development of services for employers and the promotion of a person-centered approach to services for PwCDS.

	PATHWAYS RECOMMENDED ACTIONS	Level
1	**PROVIDE ACCESS OF PWCDS** to adequate and customized employment support services both at local and regional levels.	**Services, Systems**
2	**DEVELOP FLEXIBLE EMPLOYMENT RE-INTEGRATION AND SUPPORT SERVICES** (job coaching, mentoring, counselling, and peer-counselling) at all stages of employment based on a person-centred approach to meet the changing needs of PwCDs over time.	**Services, Systems**
3	**DEVELOP SERVICES TO PROVIDE INFORMATION**, help and support to employers, concerning the employment inclusion or re-integration of PwCDs.	**Services**
4	**OFFER SERVICES FOR EMPLOYERS** to manage illness-related long-term absences and return-to work challenges.	**Services**
5	**PROVIDE APPROPRIATE TRAINING FOR PROFESSIONALS in the workplaces** to facilitate return to work of PwCDs.	**Policy, Services**

7# Promote research on effective and innovative inclusion and reintegration strategies and use the available evidence for developing solutions for employment for all

PATHWAYS FINDINGS

MAPPING

- ✓ EU Pathways demonstrated that there is no adequate monitoring nor evaluation data for most of the existing strategies targeting inclusion, integration or reintegration to work for PwCDs.

EVALUATION OF EFFECTIVENESS

- ✓ Data from national reports could not be exploited to the full extend; they were heterogeneous, with complex language and mostly structured according to the information required by the contracting authority.
- ✓ Similar obstacles were identified in scientific publications; poor research methodology, large variability of outcomes and unclear definition of investigated concepts were identified as main obstacles.

ASSESSMENT OF NEEDS

- ✓ Current evidence on employment needs of PwCDs was found to be mainly focused on the negative impact of chronic health conditions on "work-functioning" or ability to work of the person and – for some diseases – evidence was extremely limited.

EVALUATION OF THE PERSPECTIVE OF STAKEHOLDERS

- ✓ There are not or many of the stakeholders don't know any specific outcome measure to evaluate effectiveness of existing strategies

	PATHWAYS RECOMMENDED ACTIONS	Level
1	**PROMOTE AGREED STUDY PROTOCOLS** and sound evaluation methods and parameters related to inclusive labour markets.	**Policy**
2	**DEVELOP and SET SPECIFIC OUTCOME MEASURES** for evaluating existing policies, systems and services related to work inclusion, integration and reintegration of PwCDs.	**Policy, Systems**
3	**CONDUCT RESEARCH ON IDENTIFYING STRATEGIES** that promote more inclusive working environments for all (i.e. identify the various employment needs of PwCDs; identify effective work reintegration strategies, identify strategies of involvement of all sectors for more inclusive policy development).	**Policy, Systems**

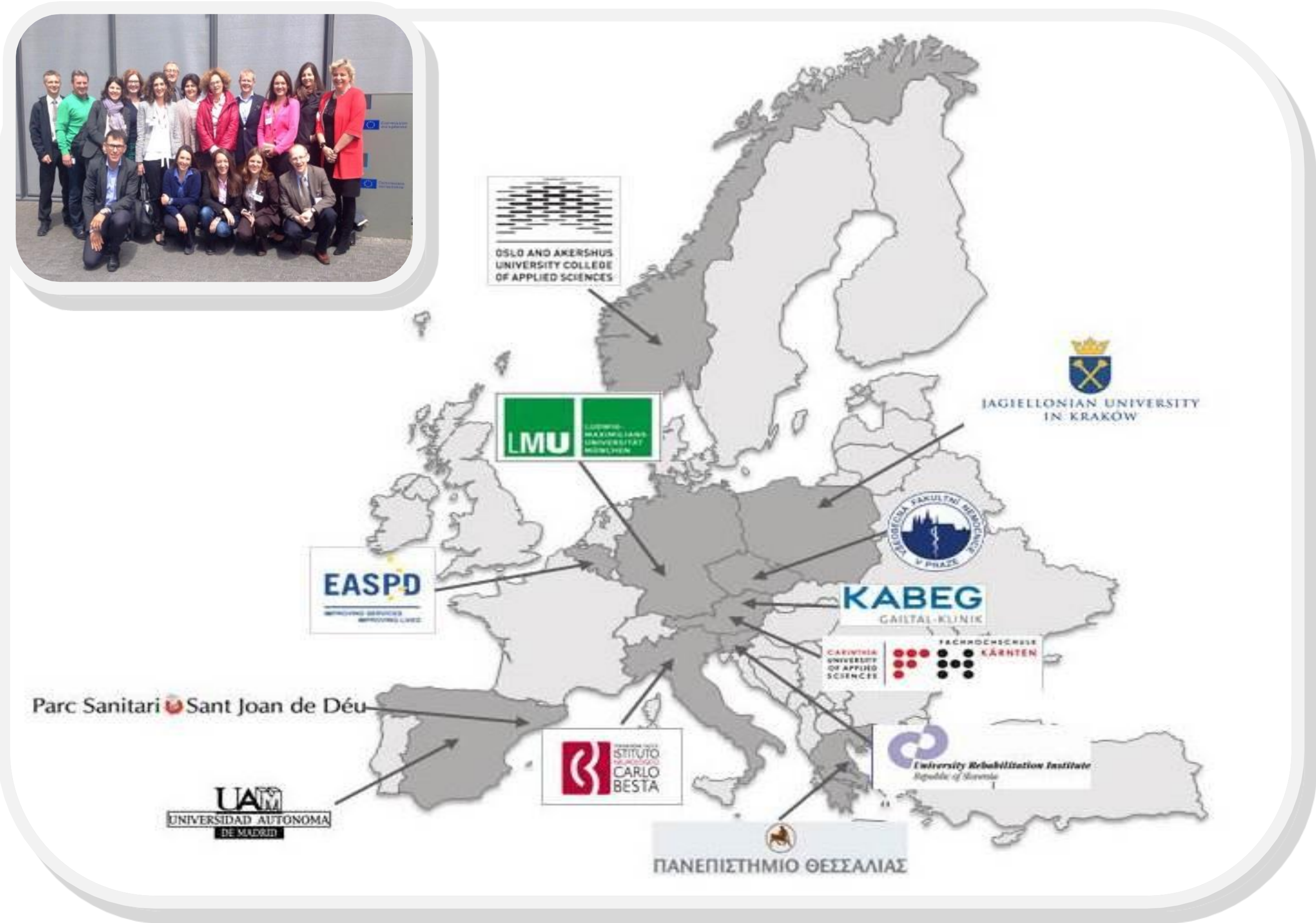

EU PATHWAYS PROJECT PARTNERS

COORDINATOR: Dr Matilde Leonardi, Fondazione IRCSS Istituto Neurologico Carlo Besta, Milan, ITALY

Universidad Autónoma de Madrid, Madrid, SPAIN

Parc Sanitari Sant Joan de Déu, Barcelona, SPAIN

Panepistimio Thessalias, Volos, GREECE

University Rehabilitation Institute, Ljubljana, SLOVENIA

Hogskolen I Oslo Og Akershus, Oslo, NORWAY

Gailtal Klinik - Neurologische Rehabilitation, Hermagor, AUSTRIA

Uniwersytet Jagiellonski, Krakow, POLAND

Ludwig-Maximilians-Universitaet Muenchen, Munich, GERMANY

Vseobecna fakultni nemocnice v Praze, Praha, CZECH REPUBLIC

Carinthia University of Applied Sciences, Klagenfurt, AUSTRIA

European Association of Service providers for Persons with Disabilities (EASPD), Brussels, BELGIUM

EMPLOYMENT & CHRONIC CONDITIONS

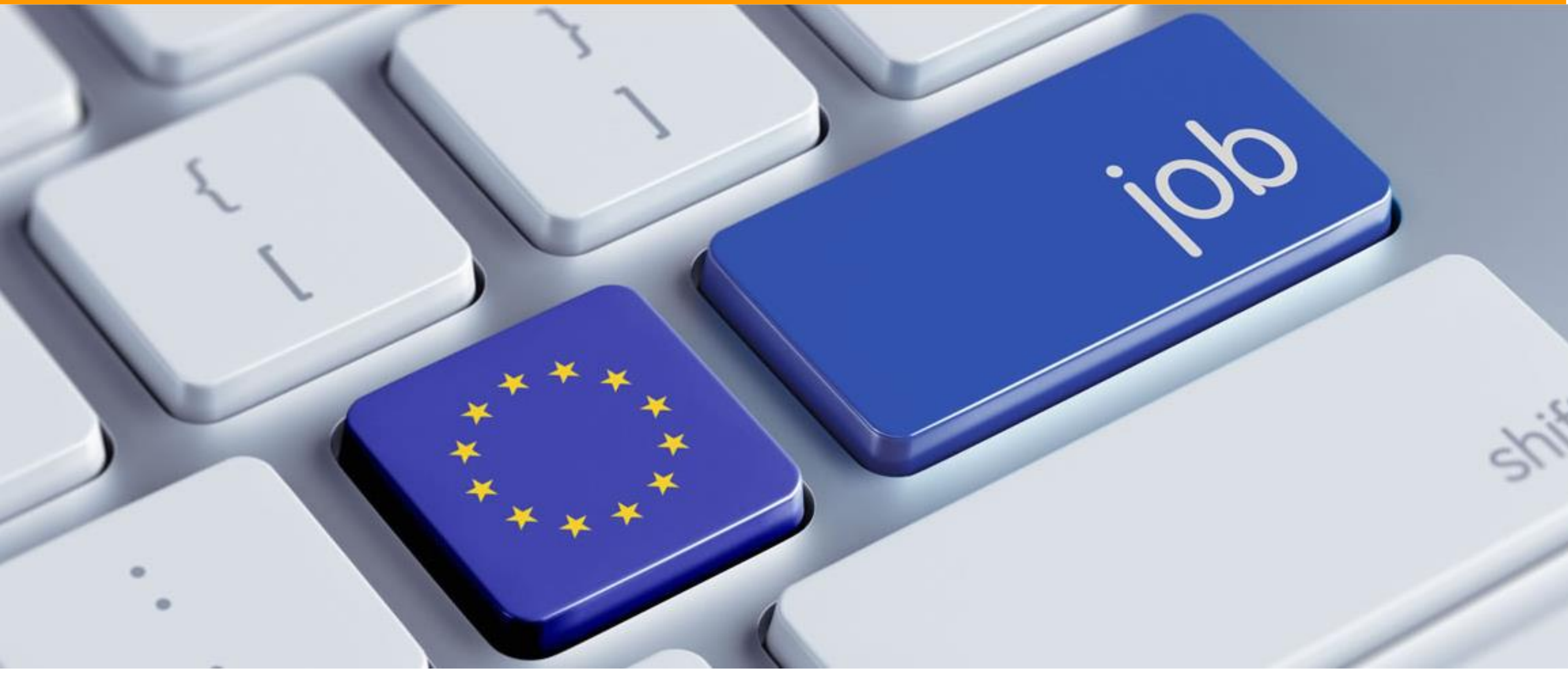

PATHWAYS PROJECT

Participation to healthy workplaces and inclusive strategies in the work sector

www.path-ways.eu

Fondazione IRCCS Istituto Neurologico Carlo Besta
Sistema Sanitario Regione Lombardia

EASPD
IMPROVING SERVICES IMPROVING LIVES

JAGIELLONIAN UNIVERSITY IN KRAKÓW

OSLO AND AKERSHUS UNIVERSITY COLLEGE OF APPLIED SCIENCES

LMU LUDWIG-MAXIMILIANS-UNIVERSITÄT MÜNCHEN

ΠΑΝΕΠΙΣΤΗΜΙΟ ΘΕΣΣΑΛΙΑΣ

CARINTHIA UNIVERSITY OF APPLIED SCIENCES
FACHHOCHSCHULE KÄRNTEN

KABEG GAILTAL-KLINIK

Parc Sanitari Sant Joan de Déu

UAM UNIVERSIDAD AUTONOMA DE MADRID

University Rehabilitational Institute Republic of Slovenia

VŠEOBECNÁ FAKULTNÍ NEMOCNICE V PRAZE

Co - funded by
the Health Programme
of the European Union
